# The expression of tenascin-C in neural stem/progenitor cells is stimulated by the growth factors EGF and FGF-2, but not by TGFβ1

**DOI:** 10.1007/s00441-021-03508-6

**Published:** 2021-07-26

**Authors:** Ursula Theocharidis, Lars Roll, Andreas Faissner

**Affiliations:** Department of Cell Morphology and Molecular Neurobiology, Ruhr University Bochum, Bochum, Germany

**Keywords:** Neural stem cells, Extracellular matrix, Growth factor, Neurospheres, EGF, FGF-2, TGFβ 1, Tenascin-C

## Abstract

Neural stem/progenitor cells (NSPCs) rely on internal and external cues determining their lineage decisions during brain development. The progenitor cells of the embryonic mammalian forebrain reside in the ventricular and subventricular zones of the lateral ventricles, where they proliferate, generate neurons and glial cells, and respond to external cues like growth factors. The extracellular matrix (ECM) surrounds NSPCs and influences the cell fate by providing mechanical scaffold, trophic support, and instructive signals. The ECM molecule tenascin-C (Tnc) is expressed in the proliferative zones of the developing forebrain and involved in the proliferation and maturation of NSPCs. Here, we analyzed the regulation of the *Tnc* gene expression by NSPCs cultivated under the influence of different growth factors. We observed that the epidermal growth factor (EGF) and the fibroblast growth factor (FGF)-2 strongly increased the expression of *Tnc*, whereas the transforming growth factor (TGF)β 1 had no effect on *Tnc* gene expression, in contrast to previous findings in cell cultures of neural and non-neural origin. The stimulation of the *Tnc* gene expression induced by EGF or FGF-2 was reversible and seen in constantly treated as well as short term stimulated NSPC cultures. The activation depended on the presence of the respective receptors, which was slightly different in cortical and striatal NSPC cultures. Our results confirm the influence of extracellular stimuli regulating the expression of factors that form a niche for NSPCs during embryonic forebrain development.

**Supplementary information:**

The online version contains supplementary material available at 10.1007/s00441-021-03508-6.

## Introduction

In the developing mammalian forebrain, neural stem/progenitor cells (NSPCs) generate the cell types of the brain in a highly ordered process, which includes the proliferation and differentiation of these cells as reviewed in several publications (e.g., Dimou and Götz 2014; Heide et al. 2017; Taverna et al. 2014). The behavior of the NSPCs and their fate decision in either direction is influenced by intrinsic and extrinsic factors (Faissner et al. 2017; Heide et al. 2017; Martynoga et al. 2012; Pacary et al. 2012; Reinhard et al. 2016; Theocharidis et al. 2014). Two subpopulations of progenitor cells can be distinguished in the proliferative zones of the mammalian forebrain that are characterized by their ability to respond to the epidermal growth factor (EGF) or fibroblast growth factor 2 (FGF-2) (Ciccolini and Svendsen 1998; Martens et al. 2000; Temple 2001; Tropepe et al. 1999). These populations exhibit self-renewal and differentiation capacity, which defines them as stem cells. The stem cell characteristics of these cells can be analyzed in vitro, where they generate free-floating cell aggregates called neurospheres when cultured in defined medium conditions and generate neurons and glia cells under differentiating conditions (Ciccolini and Svendsen 1998; Martens et al. 2000; Reynolds and Weiss 1992). The distinct populations come up in a temporally and spatially ordered fashion during development. At the beginning of neurogenesis, FGF-2-responsive cells can be found in the neurogenic regions of the forebrain, whereas the EGF-responsive populations appear later, when gliogenesis begins (Ciccolini and Svendsen 1998; Martens et al. 2000; Temple 2001; Zhu et al. 1999). The responsiveness of the NSPCs to the growth factors depends on the expression of the corresponding EGF receptor (EGFR) and FGF receptor (FGFR), which are regulated in the respective manner (Burrows et al. 1997; Zhu et al. 1999). These receptor tyrosine kinases activate intracellular signaling cascades, including the mitogen-activated protein kinase (MAPK) pathway, which influences the proliferation and differentiation of neural progenitors (Bonni et al. 1997; Campos et al. 2004; Marshall 1995; Rajan and McKay 1998). The acquisition of the EGFR depends on the activation of the FGFR and therefore succeeds the latter (Ciccolini and Svendsen 1998; Lillien and Raphael 2000; Tropepe et al. 1999). The maturation from the FGFR-expressing immature towards the more mature FGFR- and EGFR-expressing state is considerably delayed in E12.5 telencephalic and E15 spinal cord-derived NSPCs obtained from tenascin-C (Tnc) knockout tissues (Garcion et al. 2004; Karus et al. 2011). The maturation deficit could be rescued by adding purified Tnc from postnatal CNS tissue to the Tnc^−/−^-deficient NSPCs in culture (Faissner et al. 2017; Kazanis and ffrench-Constant 2011; May et al. 2018). These observations clearly suggest that Tnc as a constituent of the niche intervenes in the maturation of NSPCs as an important extrinsic factor (Faissner et al. 2017; Kazanis and ffrench-Constant 2011; May et al. 2018).

Tenascin-C (Tnc) is a multimodular glycoprotein of the extracellular matrix that is expressed during embryonic and postnatal development in different neural and non-neural tissues (Joester and Faissner 2001). During brain development, Tnc can be found in the ventricular and subventricular zones of the lateral ventricles, where it is secreted by radial glia cells (Faissner et al. 2017; Garcion et al. 2001; Gates et al. 1995; Gotz et al. 1997; Temple 2001). During human development, it shows a prominent appearance in the outer SVZ, where basal radial glia serve as neurogenic stem cells (Pollen et al. 2015). In the proliferative zone of the lateral ventricle, the number of actively cycling cells is reduced when Tnc is missing in the homozygous knockout mutant (Garcion et al. 2001). This deficit coincides with the delayed acquisition of the EGFR expression of NSPCs and the correct differentiation of neurons, astrocytes, and oligodendrocytes (Garcion et al. 2001; Garcion et al. 2004; Karus et al. 2011). During later development and in adult mice, Tnc expression can be found in zones of active neurogenesis, whereas it is downregulated in other areas (Faissner et al. 2017; Gates et al. 1995; Miragall et al. 1990). New neurons are generated in the subventricular zone of the lateral ventricle and in the dentate gyrus of the hippocampus (Bond et al. 2015; Doetsch 2003; Ihrie and Alvarez-Buylla 2011; Kempermann et al. 2004). Tnc is localized in these niches and likely influences the proliferation and fate of the stem cells (Garcion et al. 2001; Garwood et al. 2012; Gates et al. 1995; Nakic et al. 1996).

The expression of Tnc is regulated by different extrinsic and intrinsic factors, including different cytokines and growth factors (Giblin and Midwood 2015). FGF-2 and EGF provoke an induction of Tnc expression in different cell culture systems like cortical or hippocampal astrocytes or different tumor cells (DiProspero et al. 1997; Mahler et al. 1997; Meiners et al. 1993; Rettig et al. 1989; Sakai et al. 1995; Smith and Hale 1997; Wirl et al. 1995). After injection of EGF into the adult lateral ventricle, the expression of Tnc by proliferative progenitor cells with glial character is strongly increased (Doetsch et al. 2002). Another factor that shows a strong inductive effect on Tnc expression in diverse cell culture systems is the transforming growth factor (TGF) β1 (Dobbertin et al. 2010; Jinnin et al. 2004; Mackie et al. 1998; Sakai et al. 1994; Smith and Hale 1997; Wirl et al. 1995). TGFβ1 is an important regulator of neural progenitor fate and survival (Falk et al. 2008; Wachs et al. 2006) and induces matrix molecule production in pathogenic situations, which leads to the rearrangement of tissue components in the brain (Dobbertin et al. 2003; Smith and Strunz 2005). Signaling by TGFβ1 involves the activation of the TGFβ receptors 1 and 2 and is conveyed to the nucleus via Smad proteins (Massague 1987; Wrana et al. 1994). Neural stem cells of the adult neural stem cell niche are arrested by TGFβ1 treatment, but their renewal capacity or differentiation potential is not affected (Kandasamy et al. 2014; Wachs et al. 2006).

These examples illustrate that it is not possible to predict the effect of distinct cytokines on the regulation of Tnc. Rather, the cell type and the specific context determine the response that eventually results from the interaction of different signal transduction pathways. In the present study, we therefore investigated the regulation of Tnc expression in NSPC cultures by the growth factors EGF, FGF-2, and TGFβ1. We used an in vitro model to determine the proliferative capacity of different NSPC populations of the mouse embryonic forebrain and analyzed the gene expression pattern of Tnc after long- and short-term stimulation.

## Materials and methods

### Animals and tissue preparation

Time-mated female NMRI mice were obtained from Charles River Laboratories (Sulzfeld, Germany) or from the in-house animal facility. The present study was carried out in accordance with the European Council Directive of September 22, 2010 (2010/63/EU), for care of laboratory animals and approved by the animal care committee of North Rhine-Westphalia, Germany, based at the LANUV (Landesamt für Natur, Umwelt und Verbraucherschutz Nordrhein-Westfalen, D-45659 Recklinghausen, Germany). The study was supervised by the animal welfare commissioner of the Ruhr University. Mice were kept according to the German animal protection law and FELASA (Federation for Laboratory Animals Science Association) standards in a 12-h light—12-h dark rhythm with water and food ad libitum. Pregnant females were detected by the appearance of a vaginal plug. They were sacrificed by cervical dislocation, and the embryos of the gestational day 13 (embryonic day (E)13, Theiler stage 21) were removed from the uterus and their brains dissected as described earlier (Sirko et al. 2007; von Holst et al. 2007, 2006). The cerebral cortex (Cor) and the ganglionic eminences (GE) were dissected, and the meninges were removed. The tissue was enzymatically treated with 0.05% trypsin–EDTA in HBSS (Invitrogen) for 10 min at 37 °C before the addition of ovomucoid: 1 mg/ml soybean trypsin inhibitor (Sigma-Aldrich), 50 µg/ml bovine serum albumin (BSA, Sigma-Aldrich), and 40 µg/ml DNaseI (Worthington) in L-15 medium (Sigma-Aldrich). Subsequently the tissue was mechanically triturated to obtain single cell suspensions. These were centrifuged, and the cell pellets were resuspended in neurosphere medium: DMEM/F-12 1:1 (both from Sigma-Aldrich) supplemented with 0.2 mg/ml L-glutamine (Sigma-Aldrich), 2% B27 (Invitrogen), 100 U/ml penicillin, and 100 U/ml streptomycin (Invitrogen).

### Cell culture

**Fig. 1:**
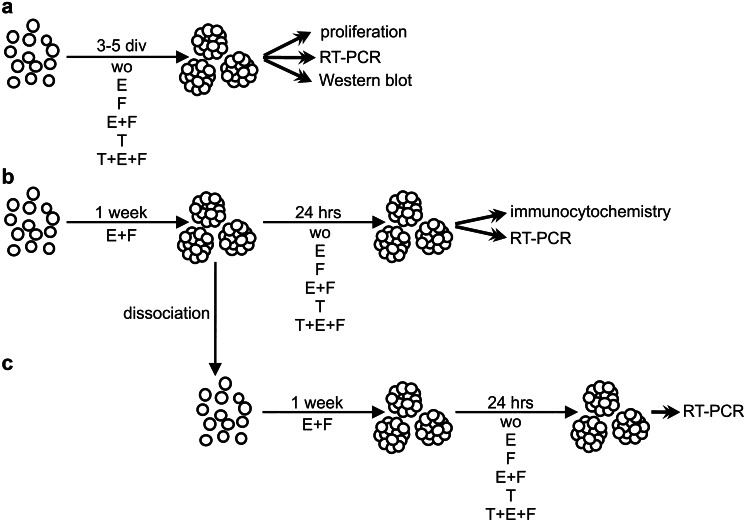
Scheme of the cell culture protocol. **a** Dissociated cells from embryonic brain tissue were cultured under defined conditions in medium containing EGF (E), FGF-2 (F), TGFβ1 (T), combinations of these factors (E + F, T + E + F) or without any additional growth factor (wo). Their proliferation capacity was determined after 5 days in vitro (div), whereas RNA and protein isolation were carried out after 3 div. **b** Cells grown in the presence of EGF and FGF-2 (E + F) generated neurospheres within 1 week. These spheres were dissociated and re-plated for the second passage (**c**). After withdrawal of the growth factors for 6 h, the neurospheres were treated with the different growth factors for 24 h and subsequently used for RNA isolation and immunocytochemistry

Dissociated cells from E13 cortical or striatal (GE) embryonic brain tissue were cultured in neurosphere medium in a density of 100,000 cells per ml in the presence of the following growth factors: EGF 20 ng/ml (E; Peprotech #100–15), FGF-2 20 ng/ml (F; Peprotech #100-18B), EGF 20 ng/ml + FGF-2 20 ng/ml (E + F), TGFβ1 10 ng/ml (T; Peprotech #100–21), TGFβ1 10 ng/ml + EGF 20 ng/ml + FGF-2 20 ng/ml (T + E + F). A previous study involving dose–response analyses had revealed robust cellular responses when the cytokines were used in these concentration ranges (Dobbertin et al. 2003). In each FGF-2 containing condition, heparin (Sigma-Aldrich #H3149) was added as co-factor in a concentration of 0.5 U/ml. Control cells were cultured without any additional growth factor (Fig. 1).

The cells were allowed to grow for 3 days in the respective medium before they were used for RNA isolation. To follow the growth over a longer period, some cultures were kept up to 5 days in vitro (div). After that time, neurospheres in 20 randomly chosen visual fields that contained a minimum of 5 assembled cells were counted. The neurosphere index was then calculated by multiplying the mean number of neurospheres (n) with a specific size (larger than 5 cells, larger than 10 (20, 50, or 100) cells) with the respective size (n1 × 5 + n2 × 10 + n3 × 20 + n4 × 50 + n5 × 100). Because under some culture conditions the cells died after the third day (visible as cell debris under the microscope), we used neurospheres grown under optimal conditions with EGF and FGF-2 for further analysis after 1 week. These cultures were transferred to growth factor-free medium for 6 h to remove the bound factors before the different growth factor combinations were added over a period of 24 h, as described above.

To assess secondary neurospheres, EGF- and FGF-2-treated primary neurospheres were collected and dissociated to single cells after 1 week using trypsin–EDTA and re-plated for second passage in the presence of EGF and FGF-2. At the end of the second week, these cells were treated with the different growth factor combinations for 24 h. The different culture and treatment routines are depicted in the schematic representation in Fig. 1.

### RNA isolation and RT-PCR

Neurosphere cells treated with different growth factors were harvested after a continuous treatment for 3 div or, alternatively, after a 24-h stimulus. The medium was removed from the cells by centrifugation, and their total RNA was isolated using the RNeasy Mini Kit (QIAGEN) following manufacturer’s instructions as described before (von Holst et al. 2007). To remove potentially contaminating genomic DNA we treated the RNA with DNase for 15 min (QIAGEN RNase free DNase Kit).

For cDNA synthesis, 1 µg of RNA was reverse transcribed using the First Strand cDNA Synthesis Kit (Fermentas/Thermo Scientific) with random hexamer primers in a total volume of 40 µl. In general, 1 µl of cDNA was used for a PCR reaction in a total volume of 25 µl using 1.5 mM MgCl_2_, 5 nmol of each dNTP, 1.25 U Taq polymerase, and 5 pmol of the appropriate forward (for) and reverse (rev) primers (Table 1). The reactions were incubated in a Mastercycler gradient (Eppendorf) with annealing temperatures as displayed in Table 1. The samples were analyzed on 1.5% agarose gels and digitally documented. Semi-quantitative endpoint analyses of the resulting band intensities were performed after background subtraction using ImageJ (NIH). Samples were first normalized to their respective *GAPDH* bands and then to the control conditions without growth factors. The statistical evaluation of three independent experiments (*n* = 3) was performed as one-way ANOVA with Bonferroni’s multiple comparison post hoc test using GraphPad Prism5 software.

**Table 1:** Primers for RT-PCR. Primers for the depicted genes were used with the shown annealing temperatures and cycle numbers. The underlined sequence parts show restriction enzyme binding sites

Primer name	Gene (accession number)	Sequence (5´-3´)	Annealing temperature	Cycle number	Product size
EGFR for	*egfr* NM_207655	ACCTCCAAGCAGTGAGTTTA	60 °C	35	554 bp
EGFR rev		TGTACAAGTGTGGCCTGCTC			
FGFR2 for	*fgfr2* NM_010207	CAGGGGACGATTCTGTGTTT	60 °C	35	356 bp
FGFR2 rev		CAGCATACATGGTGGGTCAG			
TGFβR1 for	*tgfbr1* NM_009370	GGTCTTGCCCATCTTCACAT	60 °C	35	315 bp
TGFβR1 rev		AGAACAGCGTCGAGCAATTT			
TGFβR2 for	*tgfbr2* NM_009371	ATGAGCAACTGCAGCATCAC	60 °C	35	310 bp
TGFβR2 rev		TGACACCCGTCACTTGGATA			
TGFβR3 for	*tgfbr3* NM_011578	CGGAGTACCTTCAACCCAAA	60 °C	35	312 bp
TGFβR3 rev		TGGTCACTGTCATGGATCGT			
Tnc for	*tnc* (*) NM_001369211	GCTCTAGAGGACTCCTGTACCCATTCC	60 °C	30	677 bp
Tnc rev		CGGGATCCCCAGATTTCGGAAGTTGCT			
GAPDH for	*gapdh* NM_001289726	ACTCCACTCACGGCAAATTC	60 °C	30	370 bp
GAPDH rev		CCTTCCACAATGCCAAAGTT			
β-Actin for	*actb* NM_007393	TATGCCAACACAGTGCTGTCTGGTGG	60 °C	25	247 bp
β-Actin rev		AGAAGCACTTGCGGTGCACGATGG			

### Immunocytochemical staining

After a 24-h stimulus with the different growth factor combinations, some neurospheres were dissociated to single cells and plated onto poly-Ornithin (p-Orn, Sigma-Aldrich, 10 µg/ml)-coated cell culture dishes for 3 h. The cells were washed with Krebs–Ringer-HEPES (KRH: 125 mM NaCl, 4.8 mM KCl, 1.3 mM CaCl ⋅ 2H_2_O, 1.2 mM MgSO_4_ ⋅ 7H_2_O, 1.2 mM KH_2_PO_4_, 5.6 mM D-Glucose, 25 mM HEPES, pH 7.3) and incubated with a polyclonal anti-Tnc antibody (rabbit, batch KAF14 (Faissner and Kruse 1990), diluted 1:250 in KRH/A (KRH with 1% (w/v) BSA (Carl Roth)) for 25 min before fixation with 4% (w/v) paraformaldehyde (PFA). The cells were washed with PBS/A (phosphate-buffered saline (PBS, consisting of 137 mM NaCl, 3 mM KCl, 6.5 mM Na_2_HPO_4_ ⋅ 2H_2_O, 1.5 mM KH_2_PO_4_, pH 7.3) with 1% (w/v) BSA (Carl Roth)) and incubated with a Cy2-coupled anti-rabbit secondary antibody and Hoechst/bisbenzimide (1:10^5^ in PBS/A) nuclear counterstain for 25 min. After washing with PBS/A and PBS, the dishes were mounted in PBS/glycerol (1:1) and documented using a fluorescence microscope (Axiophot 2, Zeiss).

### Western blots

Neurosphere cells cultured with different growth factor combinations for 3 div were harvested by centrifugation and lysed in lysis buffer (50 mM Tris/Cl, pH 7.4, 150 mM NaCl, 5 mM EDTA, 5 mM EGTA, 1% (v/v) Triton-X100, 0.1% (w/v) deoxycholate, 0.1% (w/v) sodium dodecyl sulfate (SDS), 40 mM sodium fluoride, 1 mM orthovanadate, pH 10) supplemented with the protease inhibitors PMSF (1 mM, MP Biomedicals), IAA (18.5 µg/ml, Sigma-Aldrich), SBTI (10 µg/ml, Sigma-Aldrich), Aprotinin (10 µg/ml, Sigma-Aldrich), Leupeptin (0.5 µg/ml, Sigma-Aldrich), and Pepstatin (0.1 µg/ml, Sigma-Aldrich) for 30 min on ice before the debris was removed by centrifugation. The conditioned medium was directly used for protein analysis. The lysates and supernatants were fractionated on a 4–10% gradient SDS polyacrylamide gel together with the Precision Plus Protein Dual Color Standard (Bio-Rad) and semi-dry blotted to methanol-activated polyvinylidene fluoride (PVDF) membranes. The membranes were blocked and incubated with antibodies for Tnc (polyclonal anti-Tnc, rabbit (batch KAF14), 1:3,000) and α-tubulin (DM1α, 1:10,000, Sigma-Aldrich), probed with appropriate HRP-coupled secondary antibodies (1:10,000, Dianova), and developed with the Clarity Western Blot ECL substrate (Bio-Rad).

### Tissue preparation for cryosections

Embryonic whole mounts were fixed in 4% (w/v) PFA overnight and subsequently cryo-protected in 20% (w/v) sucrose in PBS treated with DEPC (diethyl pyrocarbonate, 1:1,000, autoclaving after overnight incubation). After settlement the tissue was frozen in tissue freezing medium (Leica) on dry ice before the sectioning of 14-µm slices in a Leica cryostat. Sections were immediately dry-mounted and stored at – 20 °C until use.

### In situ hybridization

The protocol was adapted from Akita et al. (2008). Tissue sections were dried at room temperature and primed in 0.1 M TEA (triethanolamine hydrochloride, pH 8.0) before acetylation (0.25% (w/v) acetic anhydrate in TEA). After washing with 50 mM phosphate buffer (PB), slices were incubated for 2 h at 60 °C with hybridization buffer (50% (v/v) formamide, 10% (w/v) dextran sulfate, 1 × Denhardt’s reagent (Sigma-Aldrich), 100 µg/ml yeast RNA (Roche), 250 µg/ml salmon sperm DNA (Roche), 2 × SSC (standard saline citrate, prepared as 20 × SSC: 3 M NaCl, 0.3 M sodium citrate, pH 7.0), 50 mM sodium phosphate, pH 7.0, 0.2% (w/v) SDS). Subsequently, the sections were hybridized with a Tnc riboprobe (Czopka et al. 2009) 1:500 in hybridization buffer), which was denatured at 80 °C for 5 min before application. After overnight incubation at 60 °C, the sections were washed stringently at 60 °C in these buffers: 4 × SSC for 10 min, 2 × SSC containing 50% formamide for 20 min twice, 2 × SSC for 10 min, 0.2 × SSC for 20 min twice. The last two washing steps in Tris/NaCl buffer (0.15 M NaCl, 0.1 M Tris–HCl, pH 7.5) for 10 min were performed at room temperature, before the sections were blocked for 30 min with 1% (w/v) skimmed milk powder in Tris/NaCl buffer. Afterwards, the alkaline phosphatase-coupled anti-Dig Fab fragments (Roche) were applied (1:2,000 in blocking buffer) overnight at 4 °C. After washing with Tris/NaCl buffer thrice, the alkaline phosphatase substrates nitroblue tetrazolium (NBT, 0.34 mg/ml, Roche) and 5-bromo-4-chloro-3-indolyl phosphate (BCIP, 0.18 mg/ml, Roche) were applied in detection buffer containing 5% (w/v) polyvinyl alcohol, 0.1 M NaCl, 50 mM MgCl_2_, 0.1 M Tris–HCl, pH 9.5. The development of the color reaction was carried out at 37 °C and stopped with 1 mM EDTA, 10 mM Tris–HCl, pH 7.5 when clear signals were visible under microscopic control.

### Immunohistochemistry

Cryosections were dried at room temperature and blocked with 5% (v/v) goat serum (Jackson Immuno Research) in PBS containing 1.7% (w/v) NaCl for 1 h, before the polyclonal anti-Tnc antibody (rabbit, batch KAF14) was applied 1:300 in PBT-1 (PBS with 1% (w/v) BSA (Carl Roth) and 0.1% (v/v) Triton X-100 (Sigma-Aldrich)). After overnight incubation and washing with PBS/A, the Cy3-coupled secondary antibody (anti-rabbit Cy3, 1:500, Dianova) was added in combination with Hoechst 33258 nuclear marker (1:10^5^) in PBS/A for 2 h at room temperature. After washing with PBS, the sections were mounted with Immu-Mount (Shandon/Thermo Scientific) and documented with the Zeiss Axiophot 2.

## Results

**Fig. 2:**
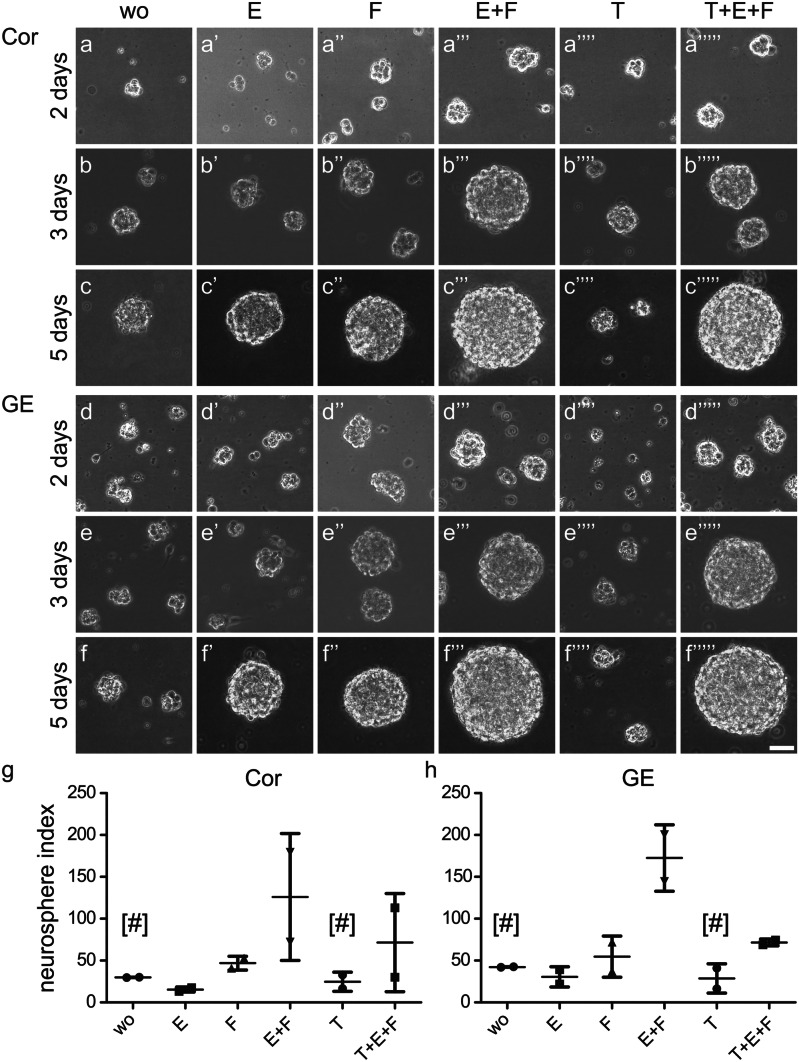
Neural stem/progenitor cells proliferated in the presence of EGF and/or FGF-2. NSPCs from the cerebral cortex (Cor, **a**–**c**’’’’’) or the ganglionic eminence (GE, **d**–**f**’’’’’) of embryonic mice grown in defined medium generated neurospheres when treated with EGF (E), FGF-2 (F), or the combination of both (E + F). The additional treatment with TGFβ1 (T + E + F) reduced the proliferation capacity of E + F-treated cells. When no growth factor was added to the medium (wo) or only TGFβ1 (T), the cells started with an initial proliferation but died between the 3rd and 5th day under these conditions [#]. The graphical representation (**g**,**h**) shows the neurosphere index representing the number and size of neurospheres in the individual cultures. Thin lines represent the median, error bars show the standard deviation (SD), and symbols represent the results of each individual experiment (*n* = 2)

Proliferation of neural stem/progenitor cells of the forebrain is dependent on the presence of growth factors. Epidermal growth factor (EGF) and fibroblast growth factor (FGF-2) stimulate the proliferation of neural progenitors in vitro, which leads to the formation of freely floating cell aggregates, the so-called neurospheres (Ciccolini and Svendsen 1998; Reynolds et al. 1992). Neural stem/progenitor cells (NSPCs) isolated at embryonic day (E)13 from the dorsal (Cor) or ventral (GE) embryonic mouse forebrain generated neurospheres in culture (Fig. 2). The resulting spheres were counted and quantified using the neurosphere index, which combines the number of neurospheres and their respective size. Depending on the culture conditions, we observed considerable variability of sphere diameters. We decided not to exclude outliers in order to avoid introducing an observer’s bias. This resulted in large error bars in some cases (Fig. 2). Control cells without growth factor support yielded few and little spheres, but most of the cells died after about 3 days in the absence of trophic support, visible as cell debris. When EGF was added to the culture medium, viable neurospheres grew to a proper size, but their numbers remained low. More neurospheres arose when FGF-2 was supplied in the culture medium. However, when both growth factors were combined, the number and size of neurospheres were larger, resulting in higher indices. This observation strongly suggests that EGF and FGF-2 synergized to drive the expansion of neurospheres. This indicates that both cytokines acted independently, possibly stimulating different fractions of progenitor cells. This may explain why the combination of both factors led to the generation of more and larger neurospheres than either of the factors alone. As remarked previously, transforming growth factor (TGFβ1) had no stimulative effect on the proliferation of neural precursors (Falk et al. 2008; Siegenthaler and Miller 2005; Wachs et al. 2006). In contrast, TGFβ1 rather reduced the proliferation rate of the NSPCs when it was added to the promotive factors FGF-2 and EGF. This was visible for cortical cells and for cells from the GE (Fig. 2). Initially, TGFβ1-treated cells started proliferation during the first 3 days, but then suddenly stopped dividing and died between the 3rd and 5th day. In this they behaved like the control cultures in the absence of growth factors, indicative of missing trophic support (Fig. 2).

**Fig. 3:**
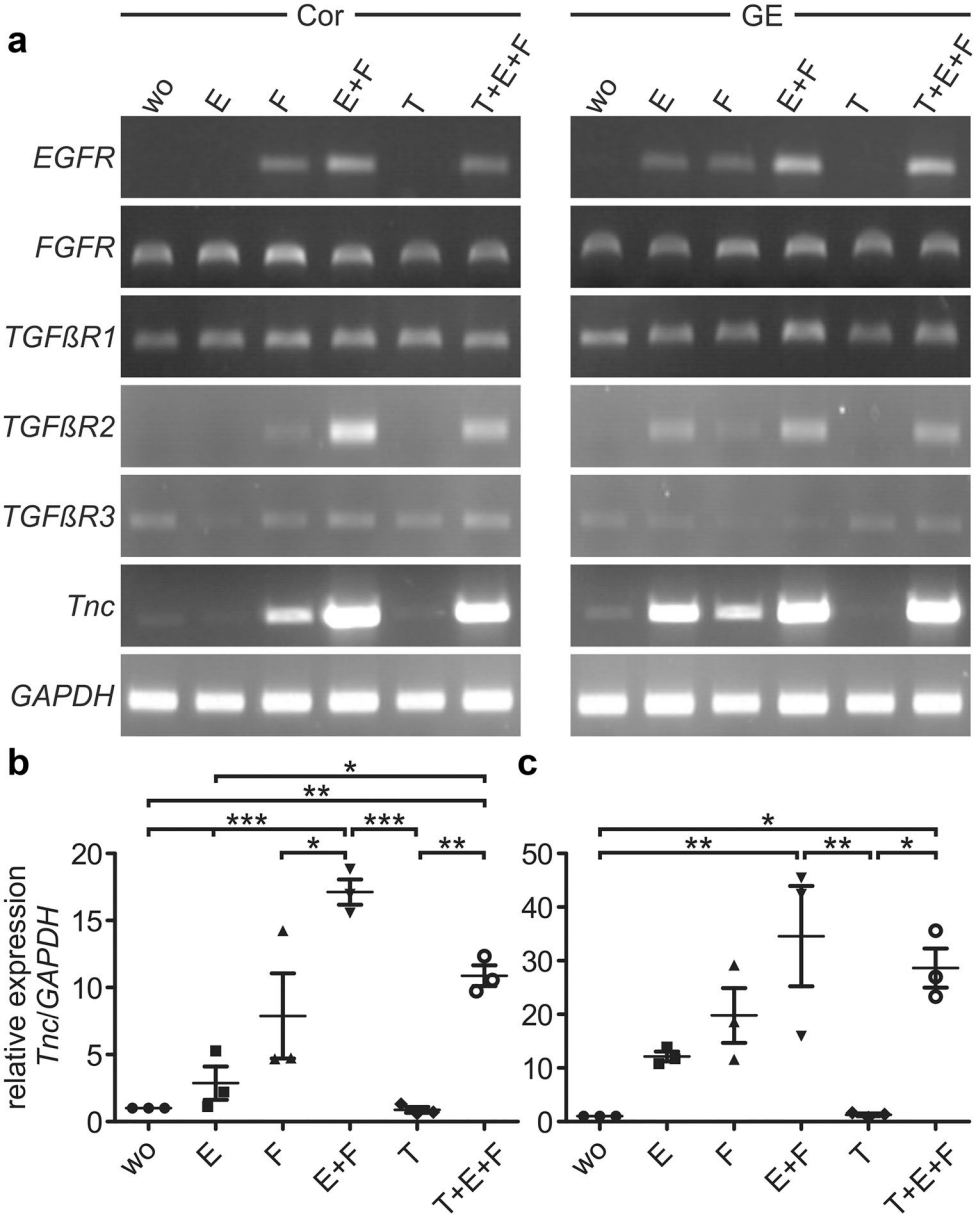
*Tnc* was upregulated in neural stem/progenitor cells by signal activation through EGF and/or FGF-2. NSPCs from the cortex (Cor) or ganglionic eminence (GE) were grown without any additional growth factor (wo), with EGF (E), FGF-2 (F), a combination of both (E + F), TGFβ1 (T), or all three growth factors together (T + E + F). **a** In cortex cells the *EGF receptor (EGFR)* was not yet found to be expressed, whereas small amounts could be detected in cells from the GE. The *EGFR* was upregulated in the presence of FGF-2. The *FGF receptor 2 (FGFR2)* was present in all cultures and therefore could mediate FGF-2 signaling to the cells. The *TGFβ receptors (TGFβR) 1* and *3* were equally expressed in the analyzed cultures, whereas the expression of *TGFβR2* depended on the regulation by FGF-2 or EGF signaling. *Tnc* was strongly upregulated when FGF-2 was added to the cultures and as soon as the EGF pathway could be activated, *Tnc* was also increased in the presence of EGF. When EGF and FGF-2 could act together, the expression of *Tnc* was even stronger and the increase was statistically significant compared to the control condition. This was the result of the semi-quantitative evaluation of *Tnc* expression in cells from the cortex (**b**) and GE (**c**). Thin lines represent the median, error bars show the standard deviation (SD) and symbols represent the results of each individual experiment (*n* = 3). *P* values: *P ≤ 0.05, **P ≤ 0.1, and ***P ≤ 0.001

After the initial growth phase of 3 days in culture, we isolated the RNA of the precursor cells to determine the gene expression levels of genes of interest under the influence of the different growth factors. The cell cultures could be analyzed using this approach because they provided sufficient amounts of sample, as documented with the reference gene *glyceraldehyde-3-phosphate dehydrogenase* (*GAPDH*), which was equally detectable in the different settings. NSPCs of the dorsal forebrain (Cor) did not express detectable amounts of EGF receptor (*EGFR*) yet (Fig. 3). In contrast, the FGF receptor (*FGFR*) was indeed present in each culture analyzed (Fig. 3) and presumably mediated the proliferative response. The neural development in the ventral forebrain appeared slightly faster than that of the dorsal forebrain, which was mirrored by the expression of the *EGFR* on the precursor cells (Ciccolini and Svendsen 1998; Martens et al. 2000). The receptor was already detectable in neurospheres from the GE. Therefore a stimulus with EGF could lead to a cellular response in this particular case. It has been demonstrated before that the presence of FGF-2 induces the gene expression of the *EGFR* (Lillien and Raphael 2000). This is in agreement with our results, because the *EGFR* was seen in all cultures where FGF-2 had been added (Fig. 3).

The expression of the *TGFβR2* required the active signaling via EGF/EGFR, which had previously been documented in other studies (Yamane et al. 2007, 2003), e.g., for corneal epithelial cells (Shu et al. 2019). When EGFR activation was combined with the addition of FGF-2, the *TGFβR2* expression was further enhanced, beyond a level obtained with FGF-2 treatment alone. This speaks in favor of a synergism of FGF-2 and EGF with regard to *TGFβR2* expression that was maintained in the presence of TGFβ1, whereas TGFβ1 by itself completely repressed *TGFβR2.* The genes of the two other known TGFβ receptors *TGFβR1* and *TGFβR3* were detectable under all analyzed conditions and did not appear regulated by any of the growth factors used in this study (Fig. 3).

**Fig. 4:**
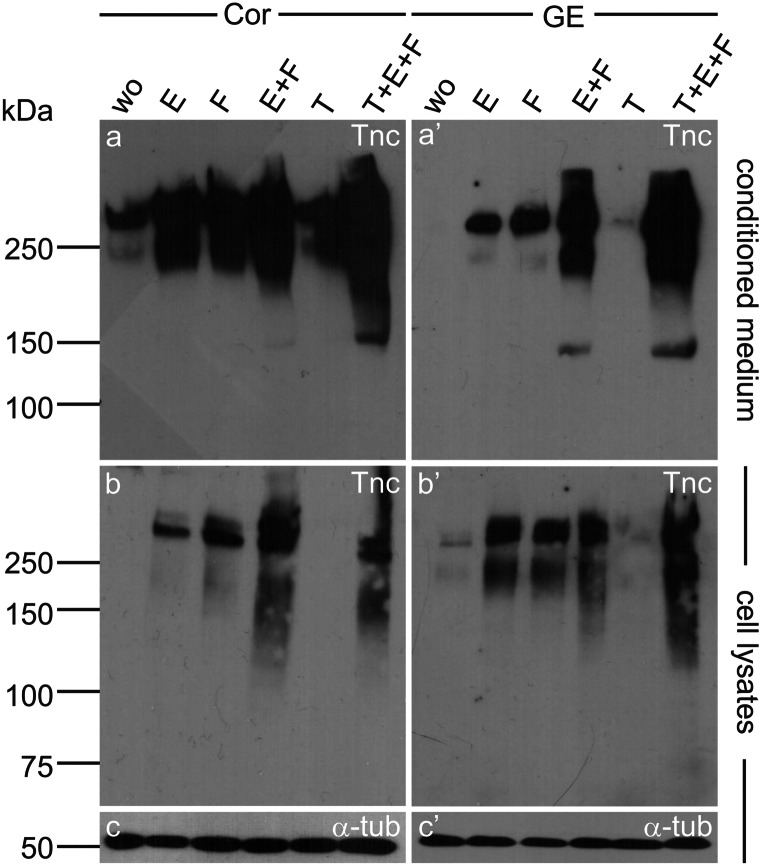
Western blot analysis of neurospheres grown under different growth factor conditions showing Tnc expression. Cortical and striatal neurosphere cells were grown in medium containing different growth factors or their combinations as depicted before. The medium supernatant was freed from any cells by centrifugation and applied to an SDS gel. **a**, **a**’ Western blotting and immunodetection with the polyclonal Tnc antibody revealed only weak signals in conditioned medium from control cells without growth factor (wo). As soon as EGF (E), FGF-2 (F), or both together (E + F and T + E + F) were present, the Tnc expression was strongly enhanced. Cultures with TGFβ1 alone showed similarly low expression levels as the controls. The expression seemed to be somewhat stronger in the cortex than in the GE but this varied among experiments. **b**, **b**’ The lower part of the figure shows the Western blot results of cell lysates, which allowed to test for the reference gene α-tubulin (**c**, **c**’). The latter was comparable for all culture conditions, whereas the Tnc protein could only be detected when EGF and/or FGF-2 were present during neurosphere growth

**Fig. 5:**
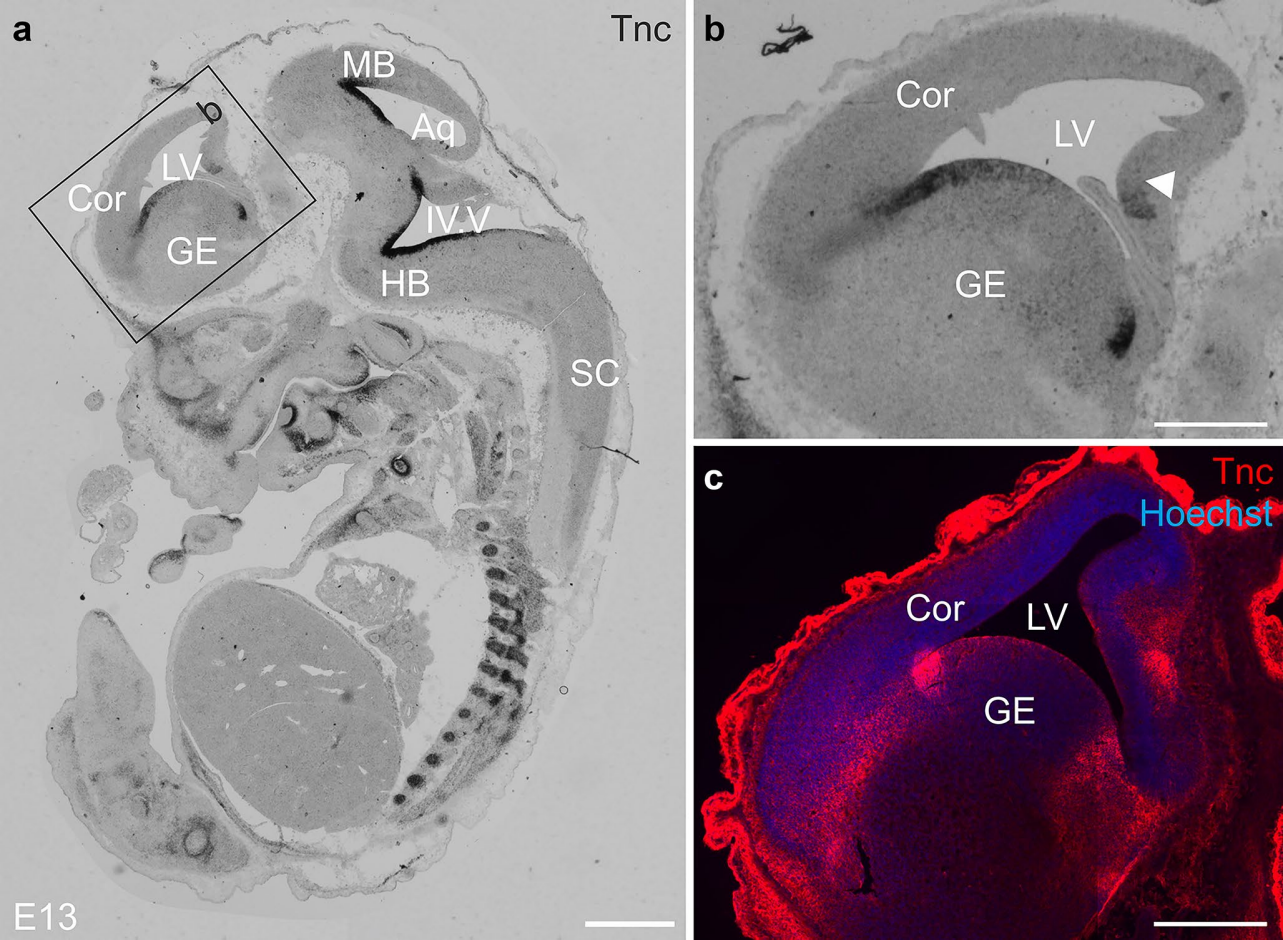
Tenascin-C expression in E13 mouse embryos. **a** Tnc was expressed in different neural and non-neural tissues as analyzed by in situ hybridizations. It could be found in the proliferative zones along the 4th ventricle (IV.V) in the hindbrain (HB) and the aqueductus mesencephali (Aq) in the midbrain (MB). In the forebrain (see larger magnification in **b**), the expression of Tnc started in the ganglionic eminence (GE) but was — apart from a small region in the hippocampal anlage — not yet present in the cortex (Cor). **c** Immunohistochemical staining for Tnc showed that the protein was localized along the lateral ventricle in the GE and spreaded to the cortico-striatal boundary. In the cortex Tnc was not yet expressed. Hoechst was used to label cell nuclei in the tissue. Scale bars: 1 mm in (**a**), 500 µm in (**b**), (**c**)

The strong regulation of the Tnc expression appeared most intriguing in our setting on mRNA level (Fig. 3) and on protein level (Fig. 4). Thus, the precursor cells from the cortex expressed only very weak levels of *Tnc* mRNA when no growth factor was present in the cultures (Fig. 3). This was expected because the expression of Tnc in situ was not yet prominent in this region at E13 (compare to Fig. 5). As soon as FGF-2 or EGF were added, the expression of the ECM molecule was strongly enhanced. FGF-2 stimulated the expression of *Tnc* in cortical cells, as well as in cultures from the GE (Fig. 3). This corresponds to the finding that Tnc expression in the ventral preceded upregulation in the dorsal forebrain and in Tnc expressing cells in the proliferative zone along the lateral ventricle in the GE (Fig. 5). In the cortex, the *EGFR* could not be found to be expressed at that early stage. *Tnc* expression in EGF-treated cortical NSPCs was negligible on mRNA level (Fig. 3). Contrarily, in ventrally derived cell cultures — where the EGFR could already be detected — the stimulation by EGF resulted in a strong increase of *Tnc* expression on mRNA level. Both growth factors combined resulted in a much stronger expression of *Tnc* in NSPCs that proved statistically significant. Also in this situation the addition of FGF-2 to EGF-responsive cells caused an augmented *Tnc* expression that exceeded notably what each factor could achieve by itself. The expression pattern of Tnc observed in vivo in E13 mouse embryos (Fig. 5) might be explained by the emergence of EGFR-positive neural progenitor cells and the subsequent activation of the intracellular signaling pathways leading to the increased transcription of the *Tnc* gene in neural progenitors of the glial lineage (Burrows et al. 1997; Temple 2001). Two days later, when gliogenesis begins at E15 and most of the progenitors in the lateral ventricular zones are responsive to EGF, the expression of Tnc expanded in the ventral as well as in the dorsal part along the whole ventricle (data not shown, and see Faissner et al. 2017).

TGFβ1 strongly promotes the expression of Tnc in numerous cell culture systems (Mackie et al. 1998; Sakai et al. 1994; Schnadelbach et al. 1998; Smith and Hale 1997; Wirl et al. 1995). As Tnc is released by NSPCs, we examined whether TGFβ1 also induces its upregulation in this context. In order to be activated by TGFβ1, the responsive cells need to express the type 2 receptor (Wrana et al. 1994). Cultures treated with a combination of EGF, FGF-2, and TGFβ1 expressed the *TGFβR2*, as detailed above. In the presence of TGFβ1, the neurosphere-forming capacity was clearly reduced. In contrast, the expression of *Tnc* was barely affected (Fig. 3). Message levels are not necessarily translated into different protein concentrations. In order to examine the consequences of factor exposure for downstream Tnc release, Western blots using the polyclonal Tnc antibody were performed (Fig. 4). The band intensities mirrored the outcome of the PCR analysis. The NSPCs secreted the ECM molecule into the medium, where it could weakly be detected in control and TGFβ1-treated cultures. EGF and FGF-2 strongly increased the amount of detectable Tnc, and the joint treatment with both growth factors exerted the most stimulatory effect on Tnc expression. The fact that EGF treatment had a slight, positive effect on neurosphere survival (Fig. 2) and on the Tnc expression, although the *EGFR* could not be detected by RT-PCR, leads to the question how EGF can exert these effects. Very low amounts of the EGFR, below the RT-PCR detection limit, might be expressed that mediate EGF signaling to a certain degree. As Tnc comprises several alternatively spliced domains (Joester and Faissner 1999; von Holst et al. 2007) and is highly glycosylated (Giblin and Midwood 2015), not one clearly circumscribed band was visible in the Western blot. Rather, Tnc glycoproteins distributed as rather diffuse bands covering a broad molecular weight range, in particular when the expression was substantial (Fig. 4).

The cell lysates (Fig. 4b, b’) also provided evidence for the regulation of Tnc protein by the growth factors, but the signals obtained were weaker. The protein fraction that was associated with the cell membranes or cell cytoplasm was less abundant than the secreted fraction. The cell lysates offered the opportunity to compare the protein amounts between the different culture conditions by comparison with the reference protein α-tubulin. The tubulin bands were similar for all cell cultures, confirming that comparable cell numbers were available in the samples. This underlined the regulatory impact of the growth factors with regard to Tnc in the samples under study (Fig. 4c, c’).

**Fig. 6:**
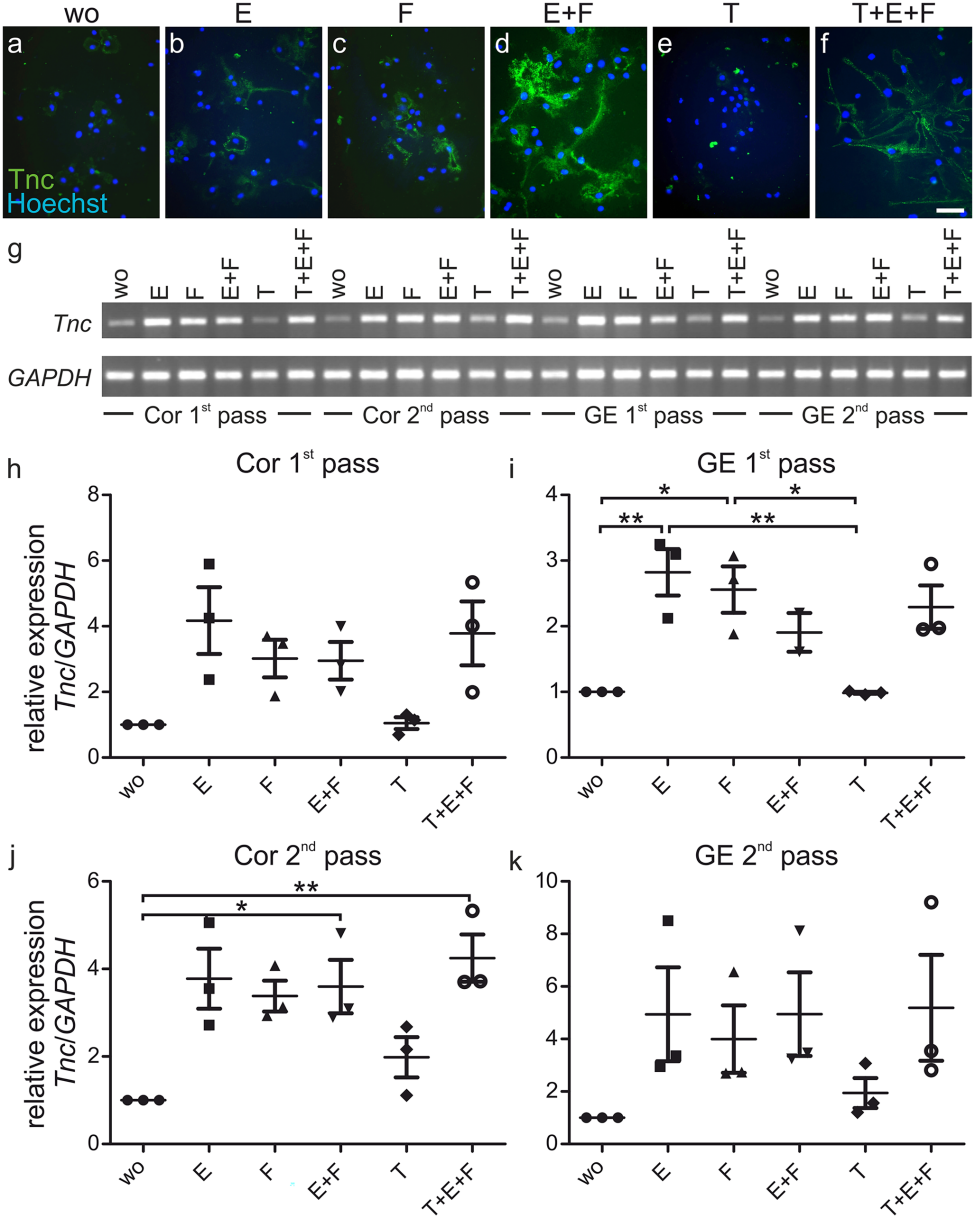
Tenascin-C expression in neurosphere cells after a short-term stimulus with growth factors. Neurosphere cells were treated with EGF (E), FGF-2 (F), TGFβ1 (T), or different combinations of these for 24 h. Control cells stayed without any growth factor (wo) during this period. **a**–**f** After plating the dissociated cells were stained for Tnc and the DNA marker Hoechst. EGF and FGF-2-treated cells immediately produced Tnc, whereas in control or TGFβ1-treated cultures the expression was weak. The combination of EGF and FGF-2 increased the Tnc expression. **g** RT-PCRs of 24-h-stimulated cortex or GE cultures of the 1st or 2nd passage showed an increase in *Tnc* expression when EGF and/or FGF-2 were present, but only low expression levels in control and TGFβ1-treated cultures (semi-quantitative evaluation in **h**–**k**). Scale bar 50 µm; thin lines represent the median, error bars show the standard deviation (SD) and symbols represent the results of each individual experiment (*n* = 3). *P* values: *P ≤ 0.05, **P ≤ 0.1, and ***P ≤ 0.001

Because NSPC cultures in media devoid of growth factors or replenished solely with TGFβ1 displayed very limited growth after the third day in vitro and failed to express *TGFβR2*, an alternative approach was used to assess their responsiveness to a short-term exposure to different growth factors. In a first step, neurospheres were generated in the presence of EGF and FGF-2 and thereafter freed of potentially bound growth factors by incubation in growth factor-free medium for 6 h. Subsequently, NSPCs were treated with different cytokine combinations over a period of 24 h (see methods scheme in Fig. 1b). This procedure tempered any effect that could result from poor cell survival or proliferation and created equal starting conditions. This method also rendered possible the generation of secondary derived from primary neurospheres maintained in the presence of EGF and FGF-2 (Fig. 1c). The number of NSPCs in secondary was higher than in primary neurospheres, which could potentially lead to different expression patterns (Reynolds and Rietze 2005; Reynolds and Weiss 1996). Cells from the cortex and the GE treated with EGF and FGF-2 for 1 week expressed the TGFβ receptors *TGFβR1*, *TGFβR2*, and *TGFβR3* (Fig. S1), which was not achieved after the shorter period of 3 days (Fig. 3). In this situation, therefore TGFβ1 could recruit TGFβR1 and TGFβR2. After 24 h we observed only faint expression of *Tnc* in control and TGFβ1-treated cultures, consistent with immunofluorescence analysis of dissociated cells and RT-PCR analysis performed with 1st and 2nd passage GE cultures (Fig. 6). Although the expression of the relevant receptors warranted the responsiveness of NSPCs, the exposure to TGFβ1 did not elevate *Tnc* expression in the first or the second passage. We conclude that TGFβ1 does not induce the expression of *Tnc* in neurosphere cells, different from what has been reported for diverse cell types of neural and non-neural origin (Mackie et al. 1998; Sakai et al. 1994; Smith and Hale 1997; Wirl et al. 1995).

In contrast, EGF or FGF-2, or both factors in combination strongly augmented *Tnc* expression in NSPCs. The protein was secreted by NSPCs that deposited a pericellular ECM in the respective environment around them, as visualized by immunostaining (Fig. 6a–f). Using RT-PCRs, we detected significantly stronger bands when the NSPCs were treated with EGF in cultures derived from the GE and also a similar tendency was seen in cells from the cortex (Fig. 6g, h–k). Because the EGF receptor was upregulated by FGF-2 in the antecedent culture condition, the difference between cortical and striatal cultures was waived. Consequently, EGF fostered a strong upregulation of the *Tnc* gene expression. FGF-2 also increased the transcription rate of *Tnc*, as expected for continuously treated cultures. Both factors led to a high expression level that reached saturation and could not be increased further, neither by the combination of both factors nor by the addition of TGFβ1 (Fig. 6). First and second passage NSPCs yielded similar results, which underlined that the stimulation of *Tnc* expression by EGF and FGF-2, but not by TGFβ1 proved a recurrent theme for these slightly different cultures. It is of interest to note that the stimulative effect by the classical growth factors of neurosphere cultures proved reversible, because cells initially treated with EGF and FGF-2 lost high *Tnc* expression when these factors were removed from the culture (Fig. 6).

## Discussion

Stem cells of the developing brain undergo symmetric and asymmetric divisions to enlarge the stem cell pool and generate differentiated cells of neural and glial lineages (Temple 2001). Along this pathway, NSPCs depend on the activity of growth factor signaling by EGF and FGF family members. When we analyzed the proliferation capacity of NSPCs in the presence of the growth factors EGF, FGF-2, and TGFβ1, we found that the progenitor cells divided frequently upon EGF and FGF-2 treatment, whereas TGFβ1 tempered their proliferation rate. The stimulation of NSPCs with EGF or FGF-2 resulted in the generation of neurospheres from embryonic forebrain tissues, in agreement with previous reports (Ciccolini and Svendsen 1998; Tropepe et al. 1999). As it had been reported for adult NSPCs, the addition of TGFβ1 led to a decreased proliferation rate of embryonic NSPCs, and the factor by itself was not able to prevent poor growth and regression of neurospheres. We assume that TGFβ1 rather arrested the stem cells in a non-proliferative, non-differentiating state, analogous to the adult neural stem cell response to TGFβ1 (Wachs et al. 2006).

Tnc is a constitutive compound of the stem cell niche in various organs (Chiquet-Ehrismann et al. 2014). Genetic transcriptome screens have revealed that Tnc is conspicuously enriched in neural stem cells in comparison with embryonic or hematopoietic stem cells (Ramalho-Santos et al. 2002). In vivo the localization of the ECM glycoprotein Tnc in the CNS coincides with the upregulation of the EGFR in the developing forebrain, which suggests that Tnc is expressed by a resident subpopulation of progenitor cells. Indeed, cells with glial characteristics produce Tnc, which has especially been shown for radial glia cells serving as glial progenitors in this case (Faissner et al. 2017). Independently of EGF, also the cultivation of NSPCs in the presence of FGF-2 led to an upregulation of *Tnc* expression, but the EGF-dependent regulation resulted in a much more prominent increase. The neurosphere cultures reproduced the correct timing of forebrain development, shown by the EGFR and FGFR expression that could be detected in a developmental stage and region-dependent manner. The pattern of expression underpinned the basis for the stimulation of *Tnc* expression by cytokines. Both receptor systems involve the MAPK pathway that fosters an increase of proliferation (Campos et al. 2004). The MAPK signaling eventually impacts gene regulation in the nucleus, where it might induce an increase of *Tnc* expression. The maturation of the stem cells reflected by the acquisition of the EGFR is promoted by Tnc in the ECM environment, as evidenced by the observation that in Tnc-deficient mice the upregulation of the EGFR is delayed (Garcion et al. 2004; Karus et al. 2011).

In the adult mouse subventricular zone, slowly dividing stem cells generate transient amplifying type C cells that are EGF-responsive (Doetsch et al. 2002). Upon exposure to EGF, the type C cells downregulate Dlx2 and start to proliferate. The cells obtained from mature mouse brain displayed pronounced Tnc expression when cultured with EGF, which suggests stimulation of Tnc expression by this growth factor. Cells from this postnatal stage do express the EGFR, which renders them independent of FGF-2 dependent growth. Consequently, the injection of EGF into the lateral ventricle of adult mice leads to the upregulation of Tnc expression by the NSPC pool, which is localized there (Doetsch et al. 2002). In our present study, we provide further evidence that the response to EGF is not confined to adult, but can also be seen in embryonic NSPCs as soon as these have acquired EGFR expression.

TGFβ1 reduced the proliferation capacity of NSPCs from the embryonic forebrain, similar to what has been reported for cultures of adult neural stem cells and astrocytes (Vergeli et al. 1995; Wachs et al. 2006). In this respect the response to this external stimulus seems comparable between the different cell culture systems. Remarkably, we observed opposing effects regarding the stimulation of Tnc gene expression by TGFβ1. The cytokine strongly upregulates Tnc in cultures of primary astrocytes (Dobbertin et al. 2010), which did not occur in our NSPC cultures. Reactive astrocytes that produce ECM components contribute a pivotal cellular component of glial scars involved in the inhibition of axonal regeneration (Fitch and Silver 2008; Vogelaar et al. 2015). TGFβ1 is an inducer of glial scar formation both in vivo and in vitro (Howe et al. 2019; Song et al. 2019). Thus, reactive glia in lesion situations reacts differently to the TGFβ1 stimulus compared to NSPCs that represent actively cycling cells involved in tissue development and remodeling. Interestingly, also the response to EGF and FGF-2 differed between NSPCs and reactive astrocytes, because the latter do not increase Tnc production upon stimulation with either growth factor (Dobbertin et al. 2010). By comparison, EGF and FGF-2 promoted a strong upregulation of Tnc in NSPCs. This might indicate that the release of Tnc into the micromilieu of the neural NSPC niche is required to maintain the stemness and proliferative capacity of this cell population. The enrichment of Tnc in stem cell niches of the CNS and other organs supports this interpretation (Chiquet-Ehrismann et al. 2014; Faissner et al. 2017). It would be interesting to examine in which way adult neural stem cells respond to TGFβ1, because they share characteristics both with embryonic NSPC as well as with astrocytes. Considering regional differences, it is conceivable that TGFβ1 leads to distinct effects in cells from the midbrain, which showed stronger responsiveness to the factor than forebrain cells, in agreement with the report that these regions also display differential responses to the factor in vivo (Falk et al. 2008).

In conclusion, Tnc is regulated by growth factors in different and occasionally even opposed ways in selected tissues, depending on the cell type and the mode of presentation. Several studies reported an increased Tnc expression when cells are treated with TGFβ1, some of them distinguishing between the distinct splice variants of the molecule (Dobbertin et al. 2010). This is clearly not the case for NSPCs. FGF-2 stimulates Tnc expression both in neural and non-neural cell cultures (Chen et al. 2009; Meiners et al. 1993). Our findings clearly demonstrate an increased expression in FGF-2-treated NSPCs. This might generate a conducive microenvironment for proliferation and differentiation of the neural stem cell pool (Faissner et al. 2017).

## Supplementary information

Below is the link to the electronic supplementary material.

Supplementary file1 (TIF 1236 KB)
