# Supplementary figures and images for: The expression of tenascin-C in neural stem/progenitor cells is stimulated by the growth factors EGF and FGF-2, but not by TGFβ1
Source: Cell Tissue Res. 2021 Jul 26;385(3):659–74. doi: 10.1007/s00441-021-03508-6 (PMC8526465; doi:10.1007/s00441-021-03508-6)

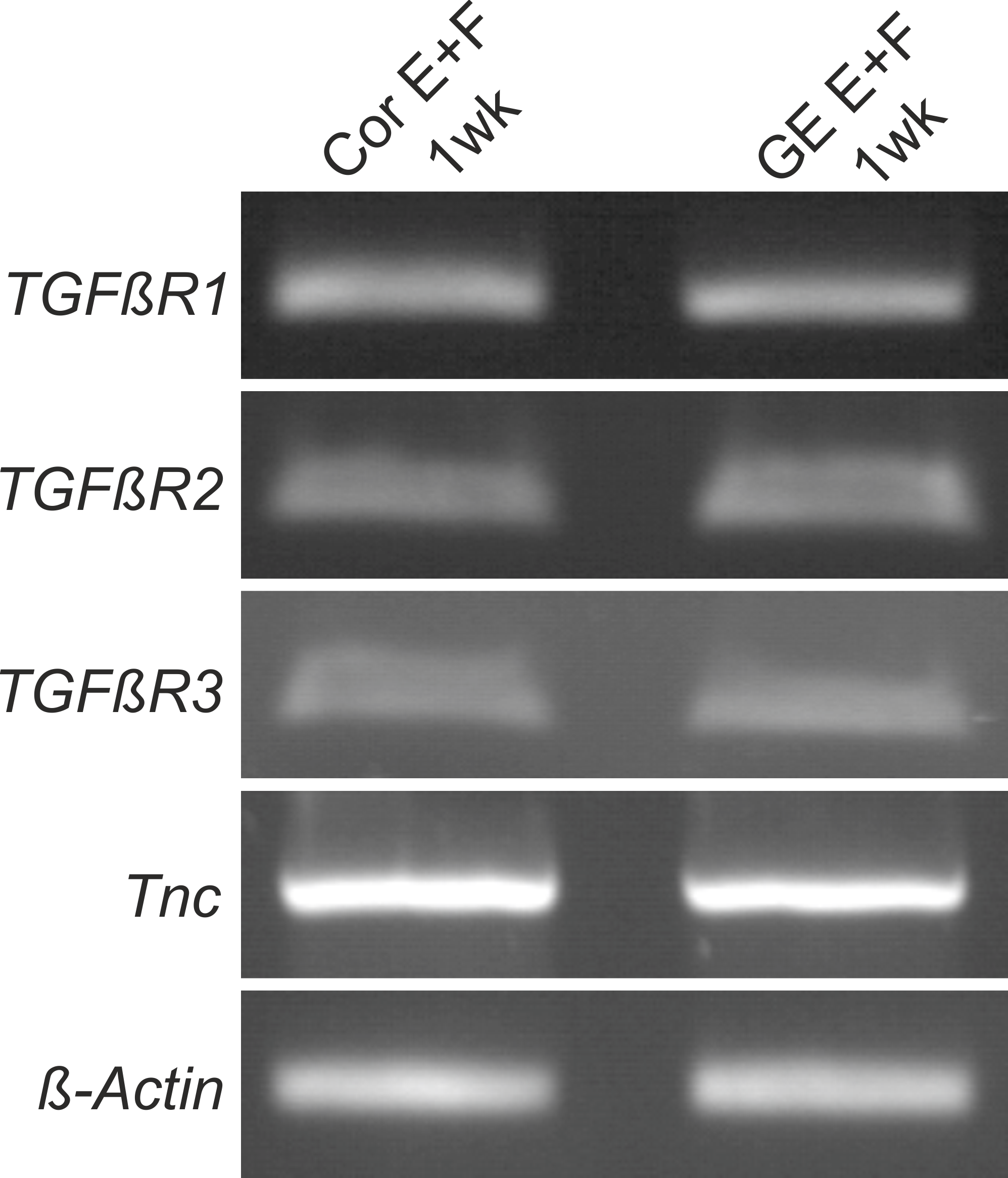

Supplement: Supplementary file 1 — Supplementary file1 (TIF 1236 KB) [file 441_2021_3508_MOESM1_ESM.tif]
